# 4D+ City Sidewalk: Integrating Pedestrian View into Sidewalk Spaces to Support User-Centric Urban Spatial Perception

**DOI:** 10.3390/s25051375

**Published:** 2025-02-24

**Authors:** Jinjing Zhao, Yunfan Chen, Yancheng Li, Haotian Xu, Jingjing Xu, Xuliang Li, Hong Zhang, Lei Jin, Shengyong Xu

**Affiliations:** 1 School of Electronics, Peking University, Beijing 100871, China; zhaojinjing@pku.edu.cn (J.Z.); 2100012767@stu.pku.edu.cn (Y.C.); lycheng@pku.edu.cn (Y.L.); xhtxys@pku.edu.cn (H.X.); 2 School of Integrated Circuits, Shandong University, Jinan 250100, China; xujj@sdu.edu.cn; 3 School of Aerospace, Beihang University, Beijing 102206, China; xulli8997@buaa.edu.cn; 4 Alpheus Robotics Technology Co., Ltd., Wuxi 214117, China; jinlei@alpheus.com.cn

**Keywords:** pedestrian-centric application, remote monitoring, computer vision, landmarks, geolocation, urban spatial perception, visualization

## Abstract

As urban environments become increasingly interconnected, the demand for precise and efficient pedestrian solutions in digitalized smart cities has grown significantly. This study introduces a scalable spatial visualization system designed to enhance interactions between individuals and the street in outdoor sidewalk environments. The system operates in two main phases: the spatial prior phase and the target localization phase. In the spatial prior phase, the system captures the user’s perspective using first-person visual data and leverages landmark elements within the sidewalk environment to localize the user’s camera. In the target localization phase, the system detects surrounding objects, such as pedestrians or cyclists, using high-angle closed-circuit television (CCTV) cameras. The system was deployed in a real-world sidewalk environment at an intersection on a university campus. By combining user location data with CCTV observations, a 4D+ virtual monitoring system was developed to present a spatiotemporal visualization of the mobile participants within the user’s surrounding sidewalk space. Experimental results show that the landmark-based localization method achieves a planar positioning error of 0.468 m and a height error of 0.120 m on average. With the assistance of CCTV cameras, the localization of other targets maintains an overall error of 0.24 m. This system establishes the spatial relationship between pedestrians and the street by integrating detailed sidewalk views, with promising applications for pedestrian navigation and the potential to enhance pedestrian-friendly urban ecosystems.

## 1. Introduction

Walking is one of the most basic and common modes of transportation. However, pedestrians are often marginalized in modern urban transportation systems, which are primarily designed for motor vehicles. This issue is especially significant for individuals with mobility impairments, older adults, children, and those with visual disabilities, who face unique challenges and increased risks in their daily movement. Sidewalks are vital spaces where these groups navigate and interact with the urban environment [1]. Improving pedestrian safety and traffic area management is essential for fostering inclusivity and promoting sustainable urban mobility.

One approach to address this issue is through intelligent transportation systems (ITS). Researchers create communication networks between users’ electronic devices and related hardware components, such as vehicle sensors, traffic sensors, and other urban infrastructure [2,3]. The timely exchange of traffic information and real-time collision warnings from approaching vehicles can be promptly delivered to pedestrians through auditory and tactile outputs. These warnings can also be converted into vehicle control signals, dynamically regulating their speeds and providing cues for pedestrians to pause and assess their surroundings [4]. Furthermore, by coordinating with infrastructure, pedestrians can receive navigation cues within designated crosswalks, allowing them to adapt to different crossing scenarios [5].

In addition to ITS, some researchers proposed to further improve pedestrians’ navigation ability and environmental perception through visual assistance technologies (VST), such as augmented reality (AR), wearable devices, and remote sighted assistance (RSA). Integrating visual sensors into these solutions can help compensate for or enhance the limitations of a user’s natural vision. New wearable electronic devices equipped with cameras extract environmental information through visual sensors and process camera images to identify features of the surroundings [6,7,8,9,10]. AR navigation tools can further enhance situational awareness and navigation efficiency by overlaying relevant information—such as directional guidance or hazard alerts—onto the user’s view of the real world [11,12,13]. RSA is another personal service that enables a blind user to establish a video connection with a remotely located sighted assistant [14]. The remote-sighted agents interpret the live video feed from the user’s device and provide real-time verbal guidance to assist via video-chat-like communication.

With the advancement of VST, vision-based pedestrian assistance systems are becoming increasingly popular for improving mobility and safety. However, using a first-person perspective comes with inherent limitations, such as a restricted field of view and reduced environmental awareness, which can often undermine the effectiveness of current assistive technologies. To address these limitations, this study explores the integration of vision-based pedestrian assistance systems with urban sensing technologies. The use of urban cameras presents a viable solution, offering advantages, such as relatively low deployment costs and extensive coverage areas. Cameras with a high-angle point of view (POV) provide superior vantage points, enabling more accurate real-time localization of users and their surrounding environments [15], including pedestrians, vehicles, and other objects. These benefits position closed-circuit television (CCTV) as a promising complement to first-person vision systems.

Building on this discussion, we propose the 4D+ city sidewalk framework. In this term, the “4D” component refers to the spatiotemporal mapping of the sidewalk environment, while the “+” signifies the system’s ability to gather information from external technologies, such as mobile devices and street cameras. This system integrates users’ visual sensing data, CCTV video, and other data sources, with a map of the sidewalk area serving as the reference base. By leveraging geolocation-based connectivity, the 4D+ framework tailors data integration to pedestrian needs and supports information visualization. Unlike existing approaches, such as outdoor scene 3D visualization that utilizes multimodal data from urban Internet of Things (IoT) deployments [16], our system emphasizes a 3D perspective derived from the synergy of data from walking users and surrounding information sources.

**Figure 1:**
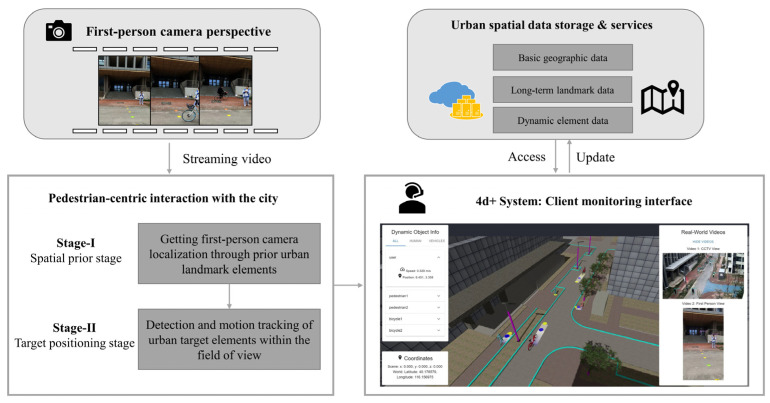
The overview of the 4D+ city sidewalk system. The system provides backend personnel with a visualization of the map and moving elements, using the user’s view and additional support from CCTV video.

To demonstrate the feasibility of the 4D+ framework, we propose a technical solution and develop a graphical interface for visualization, as presented in Figure 1. The solution integrates two types of sensors: a monocular camera from the user’s perspective and a CCTV camera with a high-angle POV. In practice, the system operates in two primary stages: the spatial prior stage and the target localization stage. In the spatial prior stage, we use predefined landmark elements, which serve as visual references within the city sidewalk map, to establish the user’s localization using the user’s first-person images. In the target localization stage, the CCTV cameras provide additional observations around the user’s surrounding environment, employing object recognition and motion tracking to localize mobile participants, such as pedestrians or cyclists. Finally, the system provides a graphical monitoring interface for backend personnel, presenting a 3D spatial representation of the user’s surroundings.

The 4D+ city sidewalk framework offers a promising solution to complement smart city planning and management efforts. One of its key strengths lies in using first-person visual sensors for pedestrian assistance, which are currently an underutilized resource. These sensors have the potential to maximize the collection of valuable sidewalk data from the user’s perspective. Recently, first-person perspective data have been increasingly valued in studies of smart city planning [17]. Advances in computer vision and wearable navigation devices are driving the integration of various visual sensing technologies for pedestrians into scalable smart city data platforms [18]. In our framework, the user’s sensing data with geolocation tags can be collected to update the urban spatial database. While they receive assistance, they simultaneously contribute data across sidewalk areas to higher-level urban management systems. This process allows pedestrian-perspective data collection to systematically capture both individual experiences and broader street dynamics, thus informing the design of more pedestrian-friendly urban environments [19,20].

The key contributions of this study are listed as follows:

- Proposes a pedestrian-centric system for enhanced walking visualization.
- Introduces a first-person vision method to enhance user positioning and utilizes CCTV for improved surrounding perception.
- Examines and validates the influence of street landmarks in a real sidewalk environment on the university campus.
- Integrates pedestrian-perspective data with maps to enhance individual–street connection.

The organization of this research is as follows. Section 2 reviews previous research on related techniques. Section 3 proposes the research and the methodological approaches in this study. Section 4 shows the results of performance evaluations and case applications based on the methods from Section 3, and Section 5 discusses further potentials and limitations of this research. Lastly, Section 6 concludes this research.

## 2. Related Work

Our work is closely related to research on providing user location information in outdoor environments and monitoring systems for walking assistance based on spatial visualization. From a technical perspective, these methods are fundamental to establishing connections between the user and sidewalk environments, serving as key components in the development of walking support systems.

To enable remote monitoring and provide additional support, pedestrian-centric systems should be capable of delivering precise first-person localization within the user’s street environment. Existing outdoor pedestrian localization methods usually obtain user location information from the Global Positioning System (GPS) [21]. As an essential component of mobile systems, GPS is a widely used method for determining a user’s location and providing directions and distances to the destination. However, GPS signals are often inaccurate, with positioning errors sometimes reaching up to several dozen meters [22]. Therefore, while GPS-based systems provide basic outdoor location information for pedestrians, researchers continue to explore methods for improving geolocation accuracy.

Previous studies explored leveraging the development of urban IoT sensors to optimize location tracking. Some studies combined GPS with specialized pedestrian positioning sensors, typically Bluetooth beacons installed within city infrastructure [23], to improve positioning accuracy. For instance, researchers [24] proposed a system that integrates the Global Navigation Satellite System (GNSS) and Bluetooth Low Energy (BLE) with smartphone inertial sensors, utilizing BLE signal transmitters installed on streetlight poles to provide accurate pedestrian location tracking in urban areas. However, they often rely on the dense installation of corresponding sensor networks.

To address this challenge, some researchers combined GPS with geospatial information, utilizing road databases, 3D building maps, and other geospatial data to optimize pedestrian positioning. For example, Weng et al. [25] leveraged pedestrian street map networks to constrain GNSS solutions. Computer vision techniques are also widely employed to assist navigation and positioning, as humans naturally recognize locations through visual cues [26,27]. One effective approach to accurately determine position in a global context is by matching current observations with prior knowledge. For example, some researchers retrieve corresponding images from a prior database to estimate the camera pose of a given query image [28,29]. Additionally, many vehicle localization studies use semantic maps to represent prior geographic data with rich semantic features of urban areas [30]. Semantic maps for automated vehicles integrate objects in driving environments, such as road markings, signs, and other structures, to enable precise localization [31,32,33], although their application in pedestrian localization research remains limited [34].

In addition to addressing the positioning issue, to obtain more diverse sensing information, researchers have made attempts to integrate multi-sensor systems with perceptive capabilities into pedestrian guidance devices, such as canes [35], guide belts [36], and guide glasses [37], to gather more diverse sensing information. The multi-sensor fusion systems mitigate the limitations of individual technologies by leveraging the complementary characteristics of various sensors, particularly in challenging environments like indoor spaces and urban canyons [38]. For instance, some intelligent canes have been developed that integrate laser and visual sensors, along with other sensors, such as ultrasound, infrared, inertial measurement units, and GPS, to aid navigation and obstacle avoidance [39]. Some researchers have explored the use of Light Detection and Ranging (LiDAR) sensors as the primary sensor in navigation systems, typically in combination with cameras and ultrasonic sensors for obstacle-detection indoors [40]. Through LiDAR sensors, these systems capture spatial information about the user’s surroundings and construct 2D maps of nearby areas for localized path guidance [41]. Additionally, some studies investigated indoor localization technology based on ultra-wideband beacons [42] and visible light positioning [43] through multi-sensor fusion.

Localization methods vary in their applicability and limitations across indoor and outdoor environments. On the one hand, urban sensor network-based localization requires additional infrastructure or a high density of sensor deployment. Meanwhile, LiDAR-based methods face challenges in improving the processing efficiency of dense point clouds in outdoor environments due to the high-dimensional and large-scale nature of LiDAR data. On the other hand, other localization technologies still require further exploration for outdoor applications, particularly to address issues related to sensor reliability, data fusion, and mutual interference. In comparison, vision-based localization methods appear to be a more suitable solution for pedestrian systems, as they offer an intuitive perception of environmental features through scene images and semantic information.

Therefore, we propose a map-based visual method for pedestrians, inspired by existing vehicle localization research, which utilizes pre-established maps to provide essential localization information [44]. Similar to semantic high-definition maps used in vehicle navigation, we use landmarks as geographic reference points, including street lamps, curbs, and other objects. While the application of semantic maps in pedestrian localization remains limited, our approach explores its feasibility in addressing user geolocation issues in sidewalk spaces. It offers an alternative solution to previous work that locates users through hand-raising behavior interactions captured by CCTV [45].

Our system enables the virtual visualization of outdoor sidewalk environments through the 4D+ map, which represents aspects that are underexplored in previous remote monitoring systems. The key challenge is the camera localization problem from the user’s perspective in sidewalk spaces. While it has been studied and validated in indoor remote assistance services [46,47,48], similar advancements are lacking in outdoor environments. For instance, researchers proposed a LiDAR-equipped smartphone application that could reposition a camera on an AR map within a controlled indoor setting [46]. Although researchers used virtual space technologies to simulate environmental experiences [49,50], with real-world environments mapped into virtual 3D spaces for algorithmic simulations [50], outdoor research still lacks effective solutions for real-world pedestrian applications in virtual sidewalk environments.

To the best of our knowledge, this research is the first to develop a pedestrian-centric visualization system for outdoor sidewalk scenes. This system integrates landmark-based pedestrian localization methods, combining the user’s perspective with road space and incorporating CCTV observations. Based on geolocation, the system aims to facilitate a two-way flow of data between the user’s device and spatial information [51,52], which not only enhances pedestrian safety but also provides significant social value for regional regulators and urban planners.

## 3. Methods

This section introduces a monocular vision-based implementation approach for the 4D+ system. Specifically, in the spatial prior stage, we use landmark elements to estimate the geospatial pose of the user’s camera, which represents the user agent. Subsequently, in the target localization phase, we locate target elements by leveraging perspective transformation relations (applicable to both the user’s camera and CCTV).

### 3.1. Spatial Prior Stage

**Figure 2:**
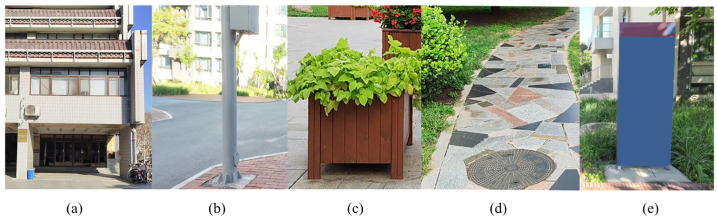
Examples of common elements in sidewalk. (**a**) buildings; (**b**) functional facilities; (**c**) vegetation landscapes; (**d**) road boundary markers; (**e**) road signage.

By leveraging landmark data, we enable the geospatial localization of the user’s camera within the sidewalk environment. In sidewalk settings, common elements, such as urban furniture, road signage, and other infrastructural components, serve as valuable references, as shown in Figure 2. When used as landmarks on the map, these urban space elements are tagged with geometric features and semantic labels.

Various types of landmark elements exhibit distinct geometric characteristics, making them suitable for spatial localization. For example, vertical features are critical for enhancing localization accuracy, while horizontal features aid in precise lateral positioning [53]. Previous studies employed landmarks, such as the vertical edges of buildings [54], streetlights [55], tree trunks [56], road boundary lines [32], and store signage [57]. While the city contains a wide range of elements, our study utilizes only a subset of these as sidewalk landmarks. In this study, we focus on a combination of tree trunks, streetlights, curbs, and manhole covers as an example of sidewalk spatial analysis.

Specifically, our method takes a first-person image frame with landmark observations and an initial coarse pose $G_{x}$ as inputs. The initial coarse pose $G_{x}=\left\{R,t\right\}$ can be manually input by the user or provided by device sensors. We adopt a six degrees of freedom (6-Dof) model to describe the camera’s position and orientation within the spatial coordinate system (i.e., the world coordinate system). The rotation matrix R∈SO(3) r represents the camera’s orientation parameters, including pitch, yaw, and roll angles, and defines the transformation from the space coordinate system to the camera’s frame of reference. The vector $t\in R^{3}$ represents the position of the camera’s optical center, indicating translation from the space’s origin to the camera’s origin.

**Figure 3:**
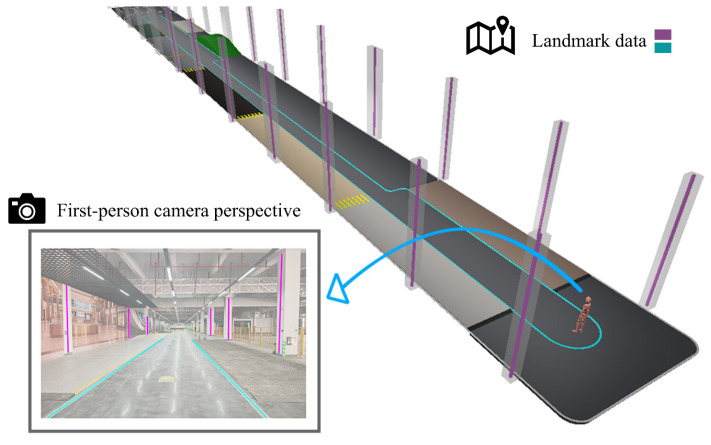
Schematic diagram illustrating the relationship between virtual landmarks (marked in purple and blue) and observations from a first-person camera view in a 3D virtual environment.

Additionally, a semantically enhanced 2.5D map is selected as a lightweight representation of landmark data, serving as prior spatial information. 2.5D maps are broadly available and easy to obtain and have already been considered for localization [58]. In this context, it refers to the representation of landmarks by ground-level shapes along with elevation property. We utilize the OpenStreetMap (OSM) XML data format to store the data [59] and manually generate the landmark locations. Figure 3 illustrates the relationship between spatial landmark elements and their projection onto the image plane of first-person camera observations.

Mathematically, we define the set of observed features *Z* at the current time t, which includes a set of observed objects $\left\{Z_{1},Z_{2},\ldots \right\}$. Each observed object $Z_{i}$ has a semantic category attribute $S\left(Z_{i}\right)$ and consists of a set of observed sample points $Z_{i}={\left\{z_{i,k}\in R^{2}\right\}}_{k=1:N_{Zi}}$, where $N_{Zi}$ is the total number of sample points in $Z_{i}$. Similarly, the spatial prior information with semantic category includes the set of landmarks M. Each landmark $M_{j}$ has a semantic category attribute $S\left(M_{j}\right)$ and consists of a set of control points $M_{j}={\left\{m_{j,k}\in R^{3}\right\}}_{k=1:N_{Mj}}$. Here, $M_{j}$ is defined as a geometric feature, such as road lines represented by curves composed of multiple control points or pole-like objects represented by line segments composed of two control points at the base and top.

For an observed object $Z_{i}$ and a corresponding landmark object $M_{c}$ with matching semantic categories, we define the following (1):

(1) $$d(Z_{i},M_{c})=\sum_{k=1}^{N_{Z_{i}}}\underset{m_{c}\in M_{c}}{min}∥z_{i,k}-m_{c}^{I}∥,$$

where the cost function $d(Z_{i},M_{c})$ that measures the dissimilarity between the observed object $Z_{i}$ and the landmark object $M_{c}$, based on their associated points. $N_{Z_{i}}\;$represents the total number of observation points corresponding to the observed object $Z_{i}$. The Euclidean distance $∥z_{i,k}-m_{c}^{I}∥$ measures the distance between the observation point $z_{i,k}$ and its nearest projected counterpart $m_{c}^{I}$ on the image plane.

Here, $m_{c}^{I}$ denotes the 2D projection of the landmark point $m_{c}\in M_{c}\;$under camera pose $G_{x}=\left\{R,t\right\}$, achieved by mapping the point from the 3D spatial coordinate system $R^{3}$ to the 2D image coordinate system $R^{2}$ using the pinhole camera projection model. Specifically, the projection is given by (2):

(2) $$m_{c}^{I}=\frac{1}{z}\cdot K[R|t]\cdot [m_{cX},m_{cY},m_{cZ},1{]}^{T},$$

where the variable $m_{c}^{I}$ represents the 2D coordinates of the landmark point $m_{c}\;$after it has been projected onto the image plane. The vector $[m_{cX},m_{cY},m_{cZ},1{]}^{T}$ is the homogeneous representation of $m_{c}$ in the spatial coordinate system. The extrinsic parameters, represented by the matrix [R|t], describe the camera’s orientation and position in the world. The factor 1/z normalizes the point to the camera’s normalized image plane, where z represents the depth. The matrix K is the intrinsic parameters of the camera, mapping coordinates from the camera’s normalized image plane to pixel coordinates.

In the special case where the landmark object $M_{c}$ is a line segment composed of two landmark points $m_{c,1}$ and $m_{c,2}$, the cost function (1) can be expressed as (3):

(3) $$d(Z_{i},M_{c})=\sum_{k=1}^{N_{Z_{i}}}d\left(z_{i,k},\;\mathrm{Line}\left(m_{c,1}^{I},m_{c,2}^{I}\right)\right),$$

where $\mathrm{Line}\left(m_{c,1}^{I},m_{c,2}^{I}\right)$ represents the line segment formed by the projected points $m_{c,1}^{I},m_{c,2}^{I}$, which are the 2D projections of the 3D landmark points $m_{c,1}$ and $m_{c,2}$ onto the image plane, respectively. This distance function d calculates the Euclidean distance from the observation point to the line segment.

Then, we iteratively optimize the extrinsic parameters of the camera to minimize the sum of the cost function values. Mathematically, this nonlinear optimization problem can be solved iteratively using the Levenberg–Marquardt (LM) algorithm, yielding the minimum distance cost between the observation and the landmark, and the estimation of the camera’s geospatial pose $G_{x}$ as (4):

(4) $$G_{x}^{*}=arg\underset{G_{x}}{min}\underset{Z_{i}\in Z}{\sum }\sum_{i=1}^{N_{Z_{i}}}d(Z_{i},M_{c}),$$

where $G_{x}^{*}$ denotes the optimal estimation of the camera’s geospatial pose. The term arg⁡min represents the optimization process that seeks the minimum cost function by iteratively adjusting the camera pose $G_{x}$.

**Figure 4:**
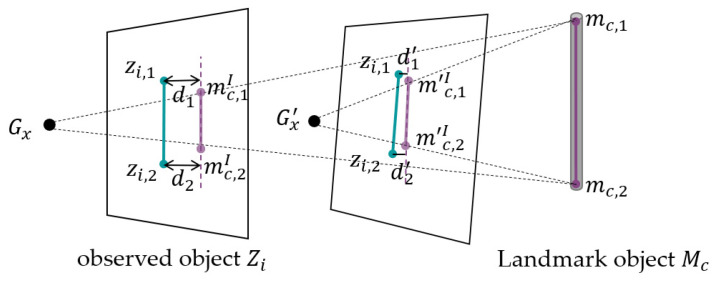
Illustration of landmark optimization and distance minimization under camera pose adjustment from $G_{x}$ to $G_{x}^{\prime }$.

To facilitate understanding, we provide a worked example with a line segment landmark object, as illustrated in Figure 4. The landmarks and observations are depicted in purple and blue, respectively. In the original camera pose $G_{x}$, the two endpoints of the landmark $M_{c}$ are represented as a line segment, denoted by $m_{c,1}^{I}$ and $m_{c,2}^{I}$ on the image plane. The $\mathrm{Line}\left(m_{c,1}^{I},m_{c,2}^{I}\right)$ forms distances $d_{1}$ and $d_{2}$ with the two points of the observation object $Z_{i}$. In the new camera pose $G_{x}^{\prime }$, the new line segment is denoted by ${m^{\prime }}_{c,1}^{I}$ and ${m^{\prime }}_{c,2}^{I}$. The optimization proceeds in the direction of minimizing the cost, as indicated by the smaller distances $d_{1}^{\prime }$ and $d_{2}^{\prime }$ under $G_{x}^{\prime }$.

During each iteration, the system evaluates the alignment of projections by the cost function and optimizes the alignment of the semantic landmark set by the camera’s extrinsic parameters. The optimized cost serves as a criterion to assess whether the current pose provides a satisfactory solution. Additionally, if the correspondence between the observation set and the landmark set is unsatisfactory, we employ a RANSAC-based method, similar to the one proposed by Xiao et al. [53], which randomly samples matching subsets within the same semantic category and evaluates their quality to select the optimal match.

At this point, we have calculated the geospatial pose of the user’s camera, allowing for the representation of the user agent in the 4D+ system. This geospatial pose establishes the transformation relationship between the first-person camera coordinate system and the spatial coordinate system. Based on this transformation, the user’s sensing data can be tagged with geolocation information for pedestrian data collection.

### 3.2. Target Localization Stage

**Figure 5:**
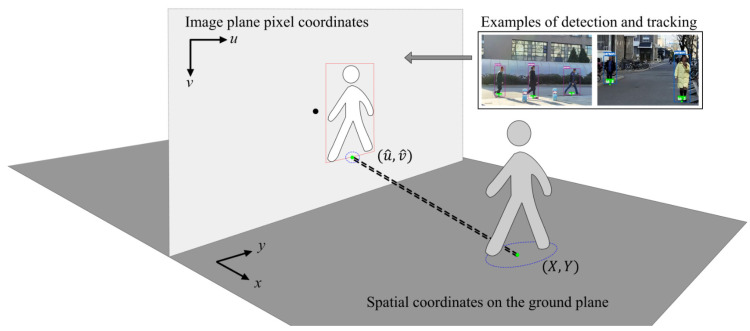
Projection transformation generates the reference point for localizing target elements in the spatial coordinate system, with examples of detection and tracking boxes from a first-person camera view.

To ensure walking safety, we focus on the user’s surrounding mobile participants, such as pedestrians and cyclists. We utilize perspective transformation relationships to localize target elements applicable to both the user’s camera and CCTV. For the input video source, we use object detection algorithms (e.g., Yolov8 [60]) combined with tracking algorithms (e.g., Deepsort [61]) to monitor moving targets (e.g., pedestrians and cyclists), as shown in Figure 5.

In the upper right-hand corner of Figure 5, we present examples captured by a monocular camera mounted on a pedestrian wearable device from a first-person perspective, with detection boxes and corresponding tracking IDs assigned to surrounding mobile participants. The detection box parameters (u,v,w,h), which include the center coordinates and dimensions, represent the target detection location in the pixel coordinates. The system assigns a unique ID to each moving target and generates continuous pixel coordinate trajectories over time.

For the detection box (u,v,w,h) of a moving target on the pixel plane, we take the pixel coordinates of the midpoint at the bottom of the box as the reference point for position conversion, representing the contact point between the central axis of the object and the ground. The reference point is expressed as (5):

(5) $$\left(\hat{u},\hat{v}\right)=\left(u,v+h/2\right),$$

where (X, Y,0) are the corresponding spatial coordinates of the object, with the *Z*-axis representing height, and the city ground plane defined as $Z_{ground}=0$. The assumption is generally reasonable, as mobile participants on sidewalks are typically ground-moving targets.

For the user’s camera view, the projection relationship between the reference point $\left(\hat{u},\hat{v}\right)$ and the position coordinates (X, Y,0) can be described by the homography matrix H as (6):

(6) $${\left[X,Y,1\right]}^{T}=H^{-1}\cdot {\left[\hat{u},\hat{v},1\right]}^{T},$$

where $H=K\left[r_{1},\;r_{2},\;t\right]$. For the CCTV view, H can be determined using single-frame annotations, as CCTV cameras are typically fixed in position.

Using the bounding box-based method, we can obtain the spatial positions of surrounding mobile participants. Subsequently, based on the user’s geolocation, we can match the mobile objects observed from CCTV to determine the user’s agent in the intersection monitoring area.

## 4. Results

**Figure 6:**
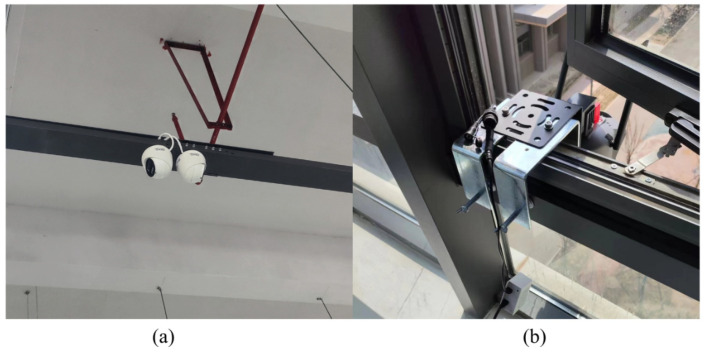
Photographs of the surveillance camera deployment for indoor (**a**) and outdoor (**b**) experiments.

We conducted three parts of testing to evaluate the target localization method, the user’s localization method, and the visualization performance of the system in the following sections. Two experimental sites with CCTV deployment were used for testing. The details of CCTV deployment are shown in Figure 6. For indoor experiments, commercial surveillance cameras were mounted at a height of 3.4 m from the ground with a 30° downward tilt, as shown in Figure 6a. In outdoor settings, cameras were installed 10 m with a 45° downward tilt, as shown in Figure 6b. For temporary mounting, a custom clamp was used to secure the camera units along window edges. All cameras featured 2560 × 1440 pixel resolution and operated at a 30 Hz frame rate.

### 4.1. Object Localization Estimation

In this section, we will test the target localization method for the proposed system, which uses monocular cameras on both user devices and CCTV. To enable efficient and fast object detection, we used the pre-trained YOLOv8n model provided by the Ultralytics library [62] and trained on the COCO dataset [63]. The training was executed under an initial learning rate of 0.01, momentum of 0.937, and weight decay of 5 × 10^−4^. Images were resized to 640 × 640 pixels. The model was trained for 100 epochs with a batch size of 16. We applied a learning rate scheduler and early stopping to ensure effective convergence and prevent overfitting. The trained YOLOv8n model achieved a mean average precision (mAP) of 37.3% on the COCO validation set.

**Figure 7:**
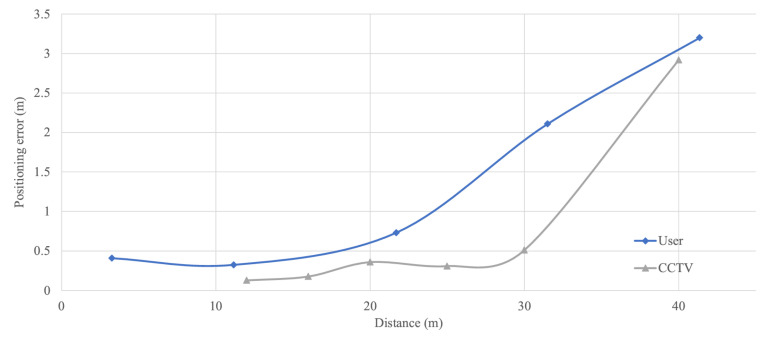
Positioning error comparison between first-person and CCTV perspectives. The curve shows that the CCTV high-angle perspective offers lower positioning error.

We conducted the test indoors, where fixed positions with intervals were used to evaluate the localization errors between the first-person and the CCTV perspectives at different distances from the target person. The results represent positioning error measurements from the first-person and CCTV perspectives, which are depicted in Figure 7 in blue and gray, respectively. The data points represent the average positioning errors obtained from multiple measurements.

The ranging results indicate that for distances under 15 m, the first-person camera maintains a ranging error of less than 0.5 m, with an overall error of 0.37 m. For distances up to 30 m, the CCTV camera also keeps the error below 0.5 m, with an overall error of 0.24 m. Additionally, the target becomes smaller on the image plane with increasing distance, which reduces detection accuracy and leads to higher location errors. Comparative analysis reveals that the CCTV high-angle perspective offers higher accuracy (overall error of 0.24 m) and a wider effective range (up to 30 m), which can improve the observation of the user and their surrounding objects. Additionally, the CCTV perspective is subject to an unavoidable blind spot, the extent of which is influenced by the angle between the line of sight and the ground.

### 4.2. Camera Geolocation Based on Landmarks

In this section, we will test the user’s static localization method using first-person images. We conducted the test outdoors, where the test area covered approximately 1.2 square kilometers of road surface on the campus. Key landmarks present in this outdoor environment included four distinct types: streetlights, tree trunks, manhole covers, and curbs. No artificial obstacles were introduced into the testing area to ensure optimal conditions for data collection. During data collection, we collect test data over 30 m in two recording sequences. The first-person perspective visual data were collected using the DJI Osmo Action 3 camera., with a resolution of 2688 × 1512 and a frame rate of 60 Hz, under well-lit weather conditions. Image frames rich in landmarks were selected as test cases, and the camera’s intrinsic parameters were pre-calibrated using the checkerboard method.

First, we trained the DeepLabv3 model for semantic segmentation to enable landmark detection in street-level imagery. The training was executed using the Mapillary Vistas Dataset [64], with an initial learning rate of 7 × 10^−3^, a momentum of 0.9, and a weight decay of 1 × 10^−4^. Images were resized to 512 × 512 pixels. The training process was divided into two phases: a frozen phase for the first 50 epochs (training only new layers) and an unfrozen phase for the remaining epochs (fine-tuning the backbone). The batch size was set to 8, with a cosine learning rate decay. An evaluation was performed every 5 epochs using Mean Intersection over Union (MIoU) to monitor model performance.

**Figure 8:**
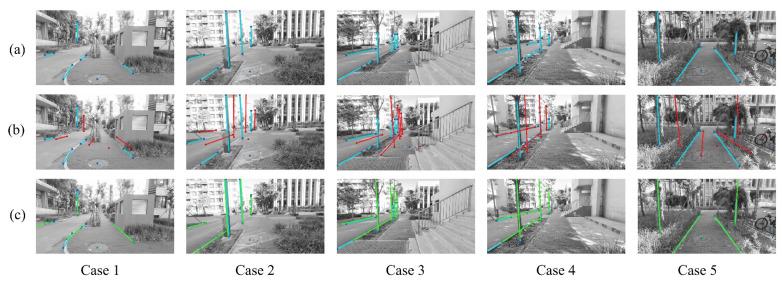
Comparative results of test cases using sidewalk landmarks for user geolocation. (**a**) test image with observed features; (**b**) projected landmark features before optimization; (**c**) projected landmark features aligned with observations after optimization.

In the experimental setting, trees, streetlights, curbs, and manhole covers recorded in the system’s landmark data were chosen as spatial priors. For the registered first-person images, the DeepLabv3 model mentioned above was employed to obtain pixel masks corresponding to the regions of the selected landmarks. Subsequently, line segment features for tree trunks, streetlights, and curbs were extracted using the principal component analysis method [65], and the center positions of manhole cover regions were calculated as point features. The detailed results for various test cases are shown in Figure 8 and Table 1. For evaluating localization errors, the ground truth camera positions are acquired using Colmap software (version 3.11) [66], a widely used structure-from-motion tool. The camera positions for each frame are geo-registered to align with the spatial coordinates. In the experiment, we add noise to the camera positions and orientation angles in the images, with the standard variances of 0.5 m and 5°.

Sidewalk landmarks provide a good alignment between the observed features with the projected landmark features, as shown in Figure 8 (see Appendix A for the detailed visualizations). In the figure, blue denotes the observed features (present in Figure 8a–c), red denotes the projected landmark features under the coarse pose (present in Figure 8b), and green denotes the projected landmark features under the precise pose after optimization (present in Figure 8c). Each column corresponds to a separate case, with five cases presented. Qualitatively, the landmark-based geolocation method reduces the pixel distances in the image plane. Additionally, we provide several quantitative metrics to evaluate the performance, as shown in Table 1.

The estimated errors for first-person geolocation are presented in Table 1, where planar positioning errors and height errors are used as the evaluation metrics. The results show that the method achieves an average planar positioning error of 0.468 m and an average height error of 0.120 m, meeting the requirements for integrating first-person visual cameras into the system. Additionally, we present the total pixel error for the best optimization of the cost function, referred to as the reprojection error. The corresponding percentage error serves as a comprehensive metric for evaluating alignment accuracy, which is calculated by dividing the reprojection error by the total image area.

The results validate that the sidewalk landmark priors from the map can accurately calculate the user’s spatial location. This capability allows the system to differentiate between the user and other objects, enabling the display of the user’s agent view in the virtual space. Furthermore, compared to lane environments, sidewalks offer a wider variety of landmark types. We also recommend combining dynamic landmarks, such as temporarily parked bicycles, with static features included in the map to enhance the robustness of the landmark feature set against potential occlusions. This strategy not only has the potential to enrich the system’s map data but also contributes to improved geospatial element management and monitoring.

### 4.3. Visualization

**Figure 9:**
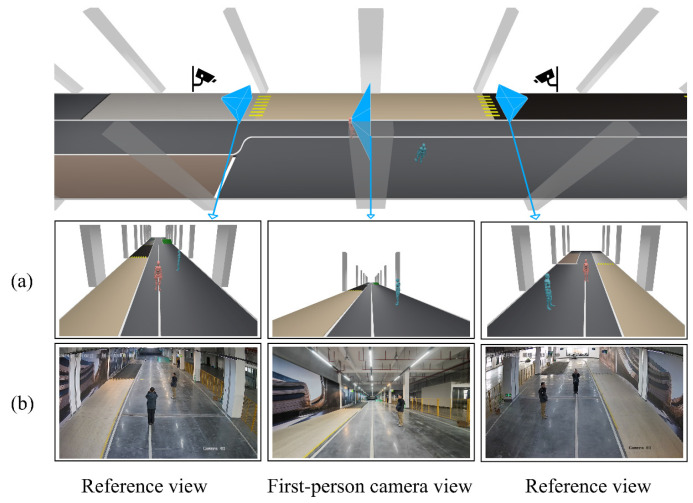
The presentation of the user and the observed pedestrian in the 4D+ system, compared with CCTV indoors. (**a**) left-side CCTV, first-person view, and right-side CCTV view in our monitoring visualization; (**b**) left-side CCTV, first-person view, and right-side CCTV images captured. The virtual perspectives are visualized in 3D space using blue frustums.

In this section, we will present the system’s visualization performance of the 4D+ virtual monitoring interface. First, we present our visualization from multiple perspectives, with indoor CCTV cameras (Figure 6a) deployed at specific perspectives. The comparisons between virtual images and real images are shown in Figure 9, including one first-person view, two CCTV views, and an overall virtual space.

As shown in Figure 9, our virtual monitoring system provides backend personnel with accurate visualizations of the user’s surrounding spatial environment. The 4D+ virtual space enables observers to perceive the local spatial structure and the positional relationships between elements more intuitively while maintaining consistency with the original images. Additionally, information privacy can be hidden from observers. This user-centered spatial perception helps support communication efficiency in human–machine interaction scenarios that require monitoring and assistance.

Next, we present the continuous virtual monitoring results within the 4D+ system. The virtual monitoring displays a representation of the user’s surrounding sidewalk space, overlaid with short-term pedestrian trajectory prediction paths for enhanced information. The system was tested outdoors (with the deployed CCTV shown in Figure 6b) at a T-shaped intersection in front of the university cafeteria.

**Figure 10:**
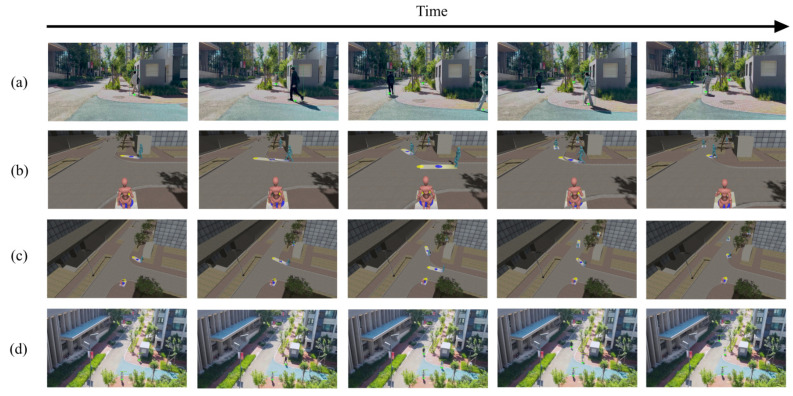
Observations of urban mobile participants in the 4D+ system over time, observed with CCTV outdoors. (**a**) the first-person view; (**b**) the frontal reference view in our monitoring visualization; (**c**) the aerial reference view in our monitoring visualization; (**d**) the CCTV view.

The spatiotemporal visualization of the mobile participants observed during the test is shown in Figure 10. The frontal and aerial reference views in our monitoring visualization consistently represent the spatial information of the real-world view within the 4D+ space. Based on the localization results from the user’s perspective, we were able to identify the user’s agent within the CCTV field of view. The user’s agent is represented by a pink virtual avatar, while other pedestrians observed from the CCTV field of view are represented by blue virtual avatars.

To alert observers of potential delays in real-world applications, we predicted the future position of the mobile participants 1.5 s and 3 s ahead based on their instantaneous speed. In Figure 10, the predicted positions at 1.5 s and 3 s are represented by purple and yellow gradients, respectively. The trajectory prediction serves as an example of basic warning signals for auxiliary applications. The visualization results demonstrate the integration of pedestrian perspectives and CCTV data into the sidewalk spatial map. Spatiotemporal reasoning and decision support information overlaid onto the virtual sidewalk spatial will help to enhance the situational awareness capabilities of observers.

## 5. Discussion

In this study, we present a 4D+ urban sidewalk navigation system that integrates monocular imagery from a first-person perspective with geospatial map data for precise localization, while utilizing overhead CCTV to enhance environmental context. The system relies on urban infrastructure elements, such as trees, curbs, lamp posts, and manhole covers, as reference landmarks, and also incorporates municipal data to detect dynamic objects like parked bicycles, improving its adaptability in complex urban environments. Experimental results show that our sidewalk landmark-based approach achieves an average planar positioning error of 0.5 m, closely aligning with typical pedestrian movement patterns. This level of precision ensures reliable navigation and pedestrian tracking within the CCTV field of view, as CCTV-based localization maintains similar accuracy.

The primary advantage of the system is its ability to improve pedestrian-centric localization by leveraging existing urban infrastructure. Unlike traditional GPS-based methods, which often face signal degradation in dense urban environments, our approach combines multiple data sources to provide more reliable positioning. By integrating both street-level and aerial perspectives, the system offers a comprehensive view of pedestrian movement and surrounding environmental conditions, making it particularly useful for urban mobility planning, enhancing accessibility, and providing guidance for pedestrians. While the system performs well for localization, traditional challenges remain, such as occlusion in crowded areas and reduced image quality in low-light or adverse weather conditions, common in vision-based localization. Hybrid sensing methods, such as infrared and stereo cameras, may help address these issues, as demonstrated in prior studies on multimodal sensing [67,68].

In addition to static localization, the system has the potential to dynamically adapt to changes in the urban environment, such as temporary blockages or altered pedestrian paths, by incorporating real-time updates of the urban spatial map. Furthermore, the system could further integrate with IoT and vehicular communication networks to enable predictive risk assessment, potentially reducing pedestrian-vehicle conflicts in high-traffic areas. For example, in the United States, pedestrian-related accidents account for approximately 16–17% of all traffic fatalities, with 84% occurring in urban environments [69]. Pedestrian safety could be improved through early warnings generated by predictive analytics in sidewalk systems, which could significantly reduce the number of pedestrian-related accidents.

On the other hand, from a technical perspective, achieving a balance between localization accuracy and energy efficiency is critical. Real-time pedestrian assistance requires continuous processing of visual data, which imposes substantial computational demands. Our initial tests show that monocular localization is effective, but long-distance travel and complex outdoor walking activities naturally increase map resource and processing requirements. Future work could explore cloud-edge computing models to reduce energy consumption while maintaining real-time performance. Additionally, using city-wide data sources, such as surveillance and traffic systems, could help offload processing from pedestrian devices, improving the overall efficiency of the system.

## 6. Conclusions

This study introduces an individual–street interaction system, which we refer to as the 4D+urban pedestrian system and its implementation. The system features a two-phase process designed to enable geospatial localization and perception from a sidewalk perspective. We connect pedestrian users with their street environment by integrating spatial information, first-person visual data, and street camera monitoring. Our system enables the virtual visualization of outdoor sidewalk environments for walking users, which represents aspects that are underexplored in previous remote monitoring systems. The experimental results validated its effectiveness in pedestrian environments and demonstrated its ability to integrate first-person views into these spaces. Although further research is necessary to improve its deployment in larger urban areas and address challenges, such as low recognition rates under extreme conditions, the system’s scalable design and ability to interface with external systems position it as a potential bridge between individual users and city sidewalk sensing networks.

## Figures and Tables

**Table 1:** Detailed results of test cases using sidewalk landmarks for user geolocation.

Test Case No.	Observed Landmarks	Reprojection Error (Pixels)	Percentage Error (%)	Planar Position Error (m)	Height Error (m)
1	Lamp pole, curb, manhole cover	22	0.73%	0.450	0.028
2	Lamp pole, curb, manhole cover	92	2.97%	0.410	0.170
3	Tree trunk, lamp pole, curb, manhole cover	110	3.60%	0.230	0.061
4	Tree trunk, curb	50	1.62%	0.600	0.170
5	Tree trunk, manhole cover, curb, temporarily parked bicycle	212	6.89%	0.650	0.170

**Figure A1:**
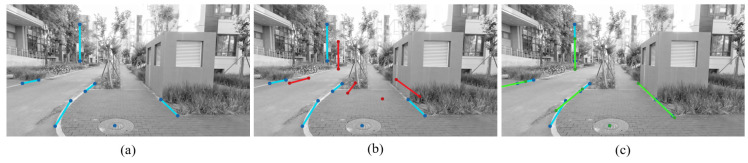
Case 1 Results.

**Figure A2:**
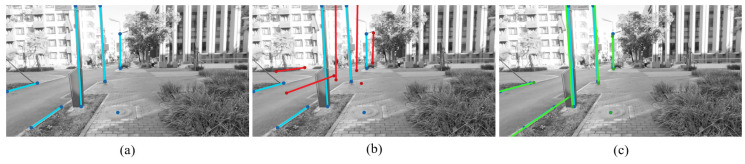
Case 2 Results.

**Figure A3:**
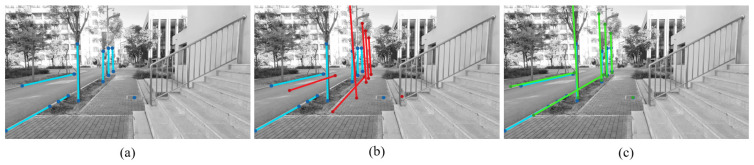
Case 3 Results.

**Figure A4:**
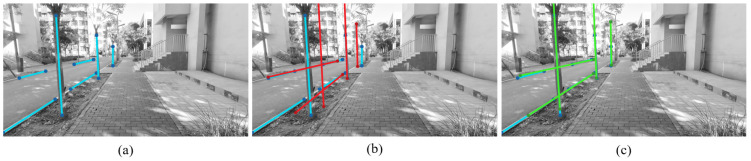
Case 4 Results.

**Figure A5:**
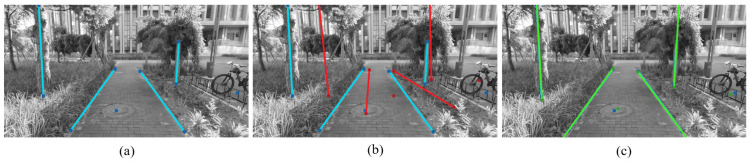
Case 5 Results.

## Data Availability

The data presented in this study are available on request from the corresponding author due to privacy reasons.

## Appendix A

We present detailed visualizations of the results for Case 1 through Case 5. To enhance clarity, we have simplified Figure 6 by splitting it into five figures. Figure A1, Figure A2, Figure A3, Figure A4 and Figure A5 correspond to the components for Case 1 to Case 5, with each figure composed of the corresponding (a), (b), and (c) subfigures in Figure 8. In these subfigures, (a) represents the original image, (b) shows the relative positions before optimization, and (c) shows the relative positions after optimization. In Figure A1, Figure A2, Figure A3, Figure A4 and Figure A5, blue denotes the observed features, red indicates the projected landmark features under the coarse pose, and green highlights the projected landmark features under the precise pose after optimization.
